# MicroRNAs as Diagnostic and Therapeutic Biomarkers in Childhood Asthma: A Systematic Review with Bioinformatics Analysis

**DOI:** 10.3390/jpm16040179

**Published:** 2026-03-25

**Authors:** Ahmed I. Alrefaey, Elena V. Vorobeva, Jamil Jubrail, Ibemusu Michael Otele, Mikaela Lee, Tilman Sanchez-Elsner, Syed Hasan Arshad, Ramesh J. Kurukulaaratchy, Mohammed Aref Kyyaly

**Affiliations:** 1 School of Technology and Maritime Industries, Southampton Solent University, Southampton SO14 0YN, UK; ahmed.alrefaey@solent.ac.uk (A.I.A.); jamil.jubrail@solent.ac.uk (J.J.); ibemusu.otele@solent.ac.uk (I.M.O.);; 2 Clinical and Experimental Sciences, Faculty of Medicine, University of Southampton, Southampton SO16 6YD, UK; e.v.vorobeva@soton.ac.uk (E.V.V.);; 3 NIHR Southampton Biomedical Research Centre, University Hospitals Southampton, Southampton SO16 6YD, UK; 4 The David Hide Asthma & Allergy Research Centre, Isle of Wight PO30 5TG, UK

**Keywords:** microRNA childhood asthma, biomarkers, diagnosis, bioinformatics, therapeutic targets

## Abstract

**Background:** MicroRNAs (miRNAs) are stable, small non-coding RNAs involved in asthma-related pathways and are promising diagnostic biomarkers and therapeutic targets in childhood asthma. **Objective:** To identify miRNAs differentially expressed in preschool wheezing and childhood asthma, evaluate their association with asthma diagnosis and severity-related phenotypes, and explore their potential translational relevance through exploratory bioinformatic analyses. **Methods:** A systematic search of Medline, Embase, SCOPUS, PubMed, CINAHL, and Web of Science was conducted for English-language articles published up to March 19, 2025. Eligible human studies reported that miRNAs were differentially expressed in children with wheeze or asthma versus healthy controls (*p* < 0.05, fold change ≥ 1.5). Bioinformatic analysis identified hub genes, constructed protein–protein interaction networks, and predicted drug–gene interactions. Results: Forty-seven studies met the inclusion criteria, yielding 58 differentially expressed miRNAs (31 up, 27 down). Recurrently reported miRNAs included miR-497, let-7e, miR-98, miR-21, miR-126a, miR-196a2, miR-1, miR-146a-5p, miR-210-3p, miR-145-5p, and miR-200c-3p across blood, nasal swabs, BALF, and exhaled breath condensate. miR-26a showed strong diagnostic performance (sensitivity 83%, specificity 93%; *p* < 0.002, 95% CI 0.831–0.987). Functional enrichment implicated 56 differentially expressed genes in metabolic and immune processes. Ten hub genes (including TNF, IL5, IL13, TLR4) were linked to 339 potential therapeutic agents; the exploratory network analysis highlighted overlap between predicted miRNA-regulated hub genes and existing asthma-relevant drug targets, including approved biologics. **Conclusions:** Our review findings suggest that several miRNAs are promising candidate biomarkers for childhood asthma phenotyping and severity assessment; however, their diagnostic utility remains exploratory and requires rigorous external validation and standardisation before clinical application.

## 1. Background

Asthma is a chronic, heterogeneous disease of the lower airways characterised by chronic inflammation and airway hyper-reactivity, leading to cough, wheeze, difficulty in breathing, and chest tightness. These symptoms can be severe and impact quality of life, particularly in children. The incidence in children is reported to be three to five times higher than in adults [1]. Diagnosing asthma in children under five years of age is particularly challenging, as similar respiratory symptoms can arise from other conditions, including viral respiratory infections, bronchiolitis, foreign body aspiration, cystic fibrosis, bronchiectasis, and tracheobronchomalacia. Clinicians primarily rely on parent-reported symptoms and opportunistic chest auscultation, methods that are subjective and limited in preschoolers [2,3]. Lung function tests and assessments of airway inflammation are frequently impractical for this age group. Consequently, there is no single gold-standard test that can definitively diagnose asthma or accurately monitor airway inflammation.

Non-invasive markers such as fractional exhaled nitric oxide (FeNO), blood eosinophils, and total serum IgE emerged as valuable biomarkers for asthma. However, their interpretation is influenced by factors including age, atopy, and corticosteroid use, limiting diagnostic specificity in childhood asthma [4]. Risk scores that combine clinical symptoms, bronchial challenge tests, and pulmonary function assessments, such as the Asthma Exacerbation Risk (AER) score, can improve risk stratification and identify children vulnerable to acute wheezing episodes, but these scores remain imperfect [5]. As a result, up to 1.2 million of the approximately 4 million people with asthma in the UK are estimated to be misdiagnosed, leading to inappropriate or incorrect medication prescriptions [6]. To address this, a diagnostic guideline was proposed by the British Thoracic Society (BTS), National Institute for Health and Care Excellence (NICE), and Scottish Intercollegiate Guidelines Network (SIGN) (https://www.nice.org.uk/guidance/ng244 (accessed on 27 November 2025)), which recommends physiological confirmation of asthma. Reliable objective biomarkers could significantly consolidate that diagnostic process, but are lacking. However, microRNAs (miRNAs) offer one potential group of biomarkers in that regard.

miRNAs are promising diagnostic biomarkers due to their regulation of immune and inflammatory pathways associated with asthma. MicroRNAs (miRNAs) are small (20 to 25 nucleotides), non-coding, single-stranded RNAs that regulate gene expression by inhibiting messenger RNA translation or inducing mRNA cleavage [7]. They are resistant to RNase activity, freely move from cells into the extracellular environment and are stable within blood, plasma, and serum [8]. Their stability improves through association with carriers like exosomes [9]. miRNA profiling enables molecular characterisation of preschool wheezing, potentially differentiating transient from persistent phenotypes and identifying children at elevated asthma risk. This offers significant clinical applicability because miRNAs can be measured from various biofluids, including blood (minimally invasive), saliva, urine, or nasal samples (non-invasive). In contrast, although fractional exhaled nitric oxide (FeNO) measurement is non-invasive, its interpretation is affected by age, atopy, and corticosteroid use, similar to blood eosinophil counts and serum immunoglobulin E (IgE) levels, limiting the accuracy of diagnosis of childhood asthma [4].

Epidemiological cohort studies have identified a range of childhood asthma phenotypes, varying from transient early wheeze to persistent severe phases. Subsequent molecular studies identified specific genetic, transcriptomic, and epigenetic biomarkers linked to asthma trajectories to distinguish phenotypes and provide mechanistic insights. In preschool children, the preferred term is “preschool wheeze” because not all early wheezers develop classic allergic asthma [10]. Endotypes reflect complex interactions among epithelial cells, smooth muscle, neutrophils, eosinophils, mast cells, and adaptive immune cells [11]. Given that many genes possess miRNA binding sites and individual miRNAs can regulate multiple targets, the miRNA regulatory network in asthma is highly complex. This complexity, combined with genetic and clinical heterogeneity, partly explains why reliable childhood asthma biomarkers and targeted therapies remain limited.

Specific microRNAs (miRNAs) are associated with asthma-related pathways: miR-200c targets interleukin-33 (IL-33), miR-23b modulates type 2 inflammation, miR-145 influences lung function, and miR-221 affects airway remodelling [12]. Additionally, miR-143-3p suppresses airway inflammation by inhibiting the pro-inflammatory transforming growth factor beta 1 (TGF-β1) signalling pathway, thereby reducing airway epithelial cell migration and suppressing lung fibroblast activity [13]. These findings indicate that miRNAs can exhibit either pro-inflammatory or anti-inflammatory roles depending on the tissue. Despite growing interest in miRNAs as asthma biomarkers, no prior systematic review has focused exclusively on miRNA expression in children, nor integrated bioinformatic analyses to map miRNA targets to phenotype-specific pathways. Furthermore, miRNA expression findings across paediatric studies remain inconsistent, due to variability in sample types, age ranges, detection methods, and diagnostic criteria. Phenotype-specific miRNA signatures that distinguish transient preschool wheeze from persistent asthma remain unclear. This review addresses these gaps by systematically identifying miRNAs differentially expressed between asthmatic or wheezing children and healthy controls, categorising them by disease severity and biological source, and using bioinformatic analysis to explore the biological relevance of candidate miRNAs through an exploratory computational analysis.

## 2. Methods

A systematic literature search was done in accordance with the Preferred Reporting Items for Systematic Reviews and Meta-Analyses (PRISMA) guidelines [14], with the completed checklist presented in Supplementary Table S2. The protocol for this systematic review and bioinformatic analysis was submitted to the International Prospective Register of Systematic Reviews (PROSPERO, Registration code: CRD420250655715). Given major clinical and methodological heterogeneity, findings were narratively synthesised with attention to sample type, phenotype category (preschool wheeze, diagnosed asthma, exacerbation), disease severity, and assay platform where reported. In addition, formal meta-analysis and publication-bias assessment were not feasible because of substantial heterogeneity in study populations, sample types, phenotype definitions, assay platforms, and outcome reporting. Likewise, a formal certainty-of-evidence framework such as GRADE was not applied; this should be considered when interpreting the findings.

We use ‘preschool wheeze’ for early-life recurrent wheezing phenotypes and reserve ‘asthma’ for studies in which a clinical asthma diagnosis was reported. Because several included studies combined these entities, interpretation of pooled narrative findings requires caution. Studies using non-specific or questionnaire-based definitions are included.

To enable valid cross-study comparison, miRNA nomenclature was standardised across studies using mature miRNA names where possible. Family-level names and strand-specific names were harmonised cautiously, and potentially equivalent entities were reviewed manually to avoid double-counting.

### 2.1. Search Strategy

A systematic search was carried out in MEDLINE, Embase, CINAHL, PubMed, Scopus, Ovid, ProQuest thesis, E-journals, and Web of Science for records published with the full text available between 1 January 2011 and 19 March 2025. The search keywords were written in a Boolean string search structure for possible synonyms alongside MeSH terms to identify articles reporting the microRNAs associated with the course of asthma from childhood until early adulthood. Complete search strategies for all databases are provided in Supplementary Table S1. The search was limited to English-language publications, which may introduce language and publication bias. This limitation potentially excludes relevant studies from non-English journals, particularly from East Asian countries, which contributed many included studies. Publication bias may also occur, as non-significant findings disproportionately appear in non-English literature. No adjustment for this bias was applied due to a lack of translation support.

### 2.2. Study Selection

Article selection was conducted by the first reviewer (A.I.A.) using a three-step process: (1) title screening, (2) abstract screening, and (3) full-text screening. Other reviewers (M.A.K., J.J., I.O., and E.V.) independently performed the same three-stage screening on all records retrieved from the database searches. Any discrepancies between reviewers were first resolved through direct discussion. Where consensus could not be reached, a third independent reviewer (J.J. or E.V.) was consulted to make the final adjudication decision.

### 2.3. Eligibility Criteria

Studies were considered eligible if they met the following criteria: (1) quantitative observational studies published in peer-reviewed journals in English; (2) the full text of the articles is available; (3) included a study population of male and female children aged under 18 years; (4) reported differentially expressed miRNA associated with wheezing, asthma diagnosis, asthma severity, or phenotype in children; and (5) applied clear and robust diagnostic criteria for moderate to severe asthma, based on one or more internationally recognised guidelines, such as BTS (British Thoracic Society), GINA (Global Initiative for Asthma), GEMA (Spanish Asthma Management Guidelines), GDPBAP (Guidelines for Diagnosis and Prevention of Bronchial Asthma in Pediatric Group), or ATS/ERS (American Thoracic Society/European Respiratory Society). Studies reporting miRNA associated with future asthma in foetal cord blood were also included.

We otherwise excluded studies that met any of the following criteria: (1) unpublished theses, master’s dissertations, reviews, clinical trials, editorials, opinion overviews, conference abstracts, or government reports; (2) studies reporting no statistically significant differences in miRNA expression; (3) studies that did not assess miRNA expression in wheezing or asthmatic children compared to non-asthmatic healthy controls; (4) studies conducted solely in vitro or using animal models; and (5) studies focused exclusively on genetic respiratory conditions, cardiovascular effects, isolated signs or symptoms, or other causes of respiratory disease unrelated to wheezing or asthma. All analyses included in this systematic review and bioinformatic analysis were based on previously published data; therefore, ethical approval and patient consent were not required.

### 2.4. Data Synthesis

Due to substantial heterogeneity in study populations (age ranges 270 days to 13 years), sample types (blood, serum, plasma, BALF, EBC, nasal lavage), detection methods (RT-qPCR, microarray, RNA sequencing), and diagnostic criteria, a formal meta-analysis was not performed. Instead, we conducted a narrative synthesis of findings with a systematic comparison of results across studies.

Among the studies included, miRNA expression was assessed using RT-qPCR, microarray platforms, or RNA sequencing. RT-qPCR was most commonly used in targeted validation studies because of its analytical sensitivity and specificity, whereas profiling approaches were used for broader discovery analyses [15] Unlike RT-qPCR, NanoString’s nCounter^®^ microRNA assay offers amplification-free, highly multiplexed counting with single-base mismatch discrimination, enabling the differentiation of homologous miRNAs. Nevertheless, it can be expensive and is sensitive to environmental factors [16,17]. Alternative detection methods, including TaqMan microRNA assays, small RNA sequencing, and GeneChip arrays, and isothermal amplification techniques such as loop-mediated isothermal amplification (LAMP) and rolling circle amplification (RCA), provide point-of-care applications such as lateral-flow tests [18,19,20,21].

### 2.5. Data Extraction

Duplicate records retrieved from the databases were removed. The full texts of the remaining references were then screened, and data were manually extracted based on the predefined inclusion and exclusion criteria. Data extraction was performed independently by the first reviewer (A.I.A.), and all extracted fields were subsequently cross-checked by a second reviewer (M.A.K.) to minimise extraction bias. Discrepancies were resolved by consulting the original study or by discussion with a third reviewer (J.J.) when necessary. The following information was collected from each study: article title, authors, publication year, study location, sample size (number of cases), child age group, sex distribution (number and percentage of male and female participants), asthma phenotypes, study setting, asthma symptoms, comorbidities, sample source, miRNA measurement techniques, specific miRNAs studied, expression levels, fold change (if reported), *p*-values, and diagnostic performance metrics such as ROC curve data, AUC (Area Under the Curve), and 95% confidence intervals (if reported). Data for both upregulated and downregulated miRNAs were presented as means, with or without standard deviations, unless otherwise stated. Outcomes were compared between asthmatic and healthy children. For targeted RT-qPCR studies, the specific candidate miRNAs analysed in each study were extracted, whereas for profiling studies, only those reported as significantly dysregulated were carried forward into the narrative synthesis. To avoid inflated overlap counts, miRNA names were harmonised prior to cross-study comparison.

### 2.6. Assessment of Study Quality and Risk of Bias

The quality of the included studies was assessed by two independent reviewers using the Quality Assessment of Diagnostic Accuracy Studies-2 (QUADAS-2) tool [22] to evaluate potential bias and study design limitations. Based on the QUADAS-2 criteria, as outlined in the guidance https://www.bristol.ac.uk/media-library/sites/quadas/migrated/documents/quadas2reportv4.pdf (accessed on 23 June 2025), studies were rated as having a “low”, “high”, or “some concerns” risk of bias.

### 2.7. Bioinformatic Analysis of miRNAs

miRNAs linked to wheezing or childhood asthma compared to healthy controls were analysed in 47 studies, using a *p*-value < 0.05 and fold change ≥ 1.5. We acknowledge that applying a nominal *p* < 0.05 threshold across many candidate miRNAs without multiple comparison correction raises the risk of false positives. To reduce this, an additional fold change threshold of ≥1.5 was applied, and for diagnostic performance analyses, a more stringent threshold of fold change ≥ 2 was used.

### 2.8. Screening of Asthma-Related Genes

Target genes related to childhood asthma were predicted from the miRNAs reported in eligible studies using the web-based programme miRWalk 3.0 (http://mirwalk.umm.uni-heidelberg.de; accessed on 29 July 2025). miRWalk 3.0 integrates five prediction algorithms—miRBase v22.1, miRDB v6.0, TarPmiR (Release 15 February 2017), TargetScan v7.2, and miRTarBase v9.0—employing machine learning models to scan the 3′ untranslated regions (UTRs) of human genes to identify seed regions complementary to mature miRNAs [23]. These databases are regularly validated through experimental methods such as reporter assays, Western blotting, microarray, and next-generation sequencing. For target prediction, only the 5′ arm of mature miRNAs was considered, and human targets predicted with greater than 90% confidence were retained. For increased prediction reliability, only genes identified as miRNA targets by at least three of the integrated databases were selected for subsequent analyses.

To verify the relevance of the screened genes to asthma pathogenesis, they were further validated using computational approaches. The GeneCards database (https://www.genecards.org) (accessed on 1 March 2026), which integrates information from over 150 web sources, was used to identify genes associated with asthma [24]. Using “asthma” as the search keyword, potential asthma-related targets were retrieved and consolidated from multiple disease-related databases within GeneCards. Duplicate entries were then removed to generate a non-redundant list of asthma-related genes for downstream analysis.

### 2.9. Functional Pathway and Enrichment Analysis

To annotate the functions of genes targeted by the identified miRNAs, the Database for Annotation, Visualisation, and Integrated Discovery (DAVID v6.8; https://davidbioinformatics.nih.gov/ (accessed on 17 March 2026) was used to perform Gene Ontology (GO) and Kyoto Encyclopedia of Genes and Genomes (KEGG) pathway analyses [25,26]. GO analysis classified target genes into biological processes, molecular functions, and cellular components, while KEGG analysis identified the regulatory pathways in which these genes are involved. The analysis was restricted to the species Homo sapiens. To account for multiple testing, both *p*-values and false discovery rate (FDR)-corrected *p*-values were computed by DAVID for all GO and KEGG pathways. Only pathways meeting both nominal significance (*p* < 0.05) and FDR < 0.05 were prioritised in the interpretation.

The online tool STRING v12.0 (https://string-db.org/) (accessed on 1 March 2026) was used to construct a protein–protein interaction (PPI) network for genes associated with childhood asthma. Interactions were retrieved using a minimum required interaction score of 0.4, and only those with a confidence score greater than 0.4 were included in the analysis. To visualise the regulatory relationships between differentially expressed miRNAs and their target genes, miRNet (https://www.mirnet.ca/) (accessed on 1 March 2026) was employed to generate a miRNA–gene interaction network.

### 2.10. Identification of Hub Genes

Protein–protein interaction (PPI) networks of asthma-related genes were visualised using Cytoscape v3.10.3. The CytoHubba plug-in (cytoHubba: identifying hub objects and sub-networks from complex interactomes) was applied to identify hub genes within the PPI network [27]. Hub gene extraction was performed using the Maximal Clique Centrality (MCC) algorithm to rank nodes by connectivity. The top 10 genes with the highest degree of connectivity were selected as hub genes. To assess the stability of hub gene identification, sensitivity analysis was performed using fold change thresholds of 1.5 and 2.0.

### 2.11. Drug–Gene Interaction

To predict potential drug candidates targeting the top ten hub genes, we employed the Drug–Gene Interaction Database (DGIdb), an online resource that links drugs or compounds to their corresponding target genes [28]. Hub genes associated with significantly expressed miRNAs in asthmatic phenotypes in children were input into DGIdb using the ‘by target gene’ search option, restricted to the species Homo sapiens. Only drugs meeting the ‘strong interaction’ criterion and having a drug–gene interaction score ≥ 0.3 were selected. The ≥0.3 threshold indicates computational predictions needing clinical validation before therapeutic use. From the resulting list, approved drugs related to the hub genes were selected as promising therapeutic candidates. Candidate drugs identified from DGIdb were further validated using the DrugBank database (https://go.drugbank.com/) (accessed on 1 March 2026) to confirm drug–gene interactions, mechanisms of action, and therapeutic indications.

## 3. Results

### 3.1. Characteristics of the Studies

**Figure 1:**
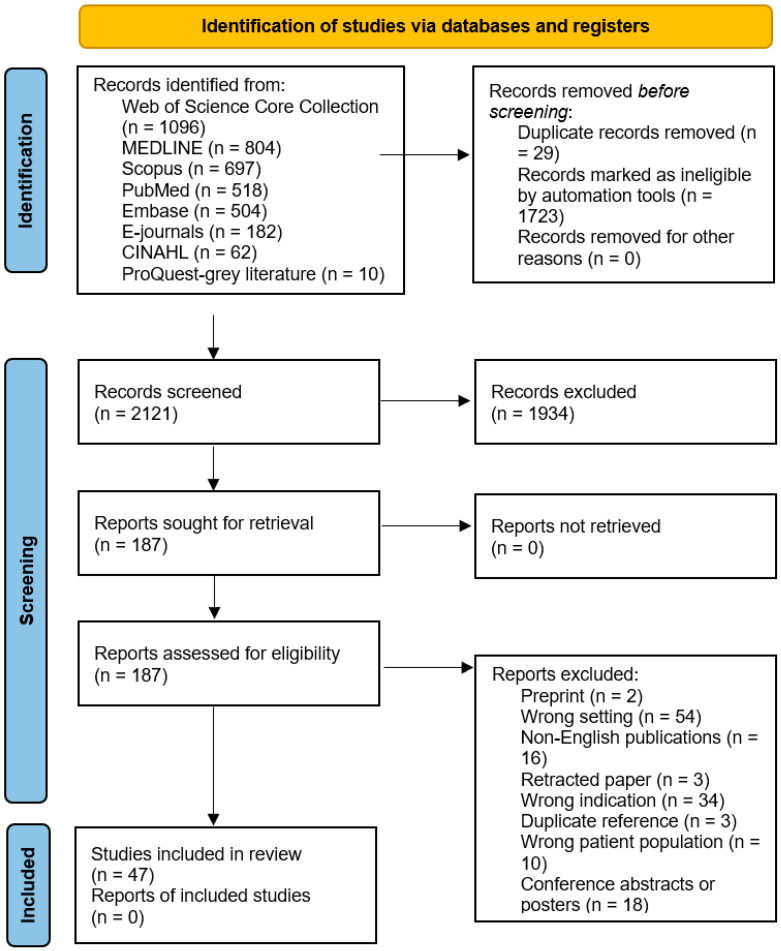
PRISMA flow diagram for study selection.

The database search yielded a total of 3873 articles. After removing duplicates, 2121 records were selected for title and abstract screening. Of these, 1934 were excluded as they were animal studies, in vitro studies, or did not meet the inclusion criteria based on setting or outcomes. To minimise publication bias, 10 additional dissertations were retrieved from the ProQuest thesis database and evaluated, but none met the eligibility criteria. A total of 187 articles were selected for full-text review, of which 47 met the inclusion criteria and were included in the review (Figure 1). These 47 studies showed substantial heterogeneity in methodology, populations, and outcome measures, precluding formal meta-analysis.

The publication year of the reviewed studies spanned from 2012 to 2025, with a clear increase in output over time: 2012 (*n* = 1); 2013 (*n* = 1); 2015 (*n* = 1); 2016 (*n* = 4); 2018 (*n* = 5); 2019 (*n* = 6); 2020 (*n* = 3); 2021 (*n* = 5); 2022 (*n* = 6); 2023 (*n* = 5); 2024 (*n* = 3); and 2025 (*n* = 2). This distribution reflects an increase in publication activity from 2018 onwards, consistent with advances in RNA sequencing technology and growing interest in miRNA biomarkers for childhood asthma. Sample sizes ranged from 10 to 555 participants, with a median sample size of approximately 120. Participants were children aged from 270 days (9 months) to 13 years, with a median age of 8.9 years across the studies (Table 1). The term preschool wheezers commonly describes children under five who have recurrent wheezing, a phenotype that may develop into persistent asthma in later childhood or resolve as transient wheeze with age. Of the 47 included studies, 2 studies were conducted on the same birth cohort, with a potential overlap of participants [29,30]. In the included studies, asthma symptoms were diagnosed either through clinical assessments during acute respiratory episodes or via parental questionnaires regarding their children’s respiratory health after birth. Five studies identified three distinct asthma/wheezing phenotypes associated with asthma outcomes in childhood: (1) persistent wheezing, occurring throughout the first six years of life and strongly linked to later school-age asthma; (2) intermittent severe wheezing; and (3) transient wheezing, observed during the first three years of life without progression to asthma in later childhood [31,32,33,34,35].

### 3.2. Quality Assessment and Risk of Bias

**Figure 2:**
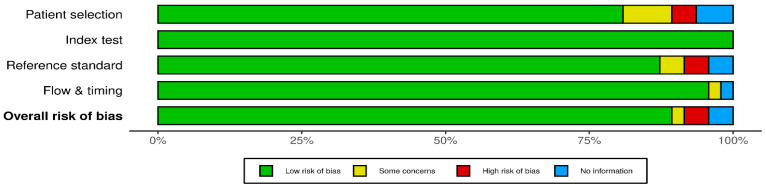
Methodological quality assessments of the included 47 articles were based on the QUADAS-2 tool. QUADAS-2 = Quality Assessment of Diagnostic Accuracy Studies–2. Overall risk of bias (shown in bold) denotes the summary risk of bias judgement derived across all four QUADAS-2 domains.

According to QUADAS-2 quality evaluation, 2 of the 47 included studies were classified as low quality [35,36]; 3 were classified as fair quality, having “some concerns” [13,37,38]; and the remaining 42 were classified as high quality (Figure 2). Although many studies fulfilled several QUADAS-2 domains, common limitations remained in patient selection, diagnostic reference standards, phenotype definition, and timing of sample collection relative to disease activity. Therefore, the overall certainty of the diagnostic evidence should be interpreted cautiously. The primary sources of bias were mainly related to study design limitations in patient selection and reference standards. Many studies selected participants based on clinical follow-up or parental questionnaire responses, often without applying robust or standardised diagnostic criteria. Asthma diagnosis techniques varied widely across studies, from lung function testing to audible wheezing or recurrent cough episodes, introducing the potential for selection bias. In some studies, the time lag between symptom onset and miRNA testing was not verified, contributing further to the risk of bias [38]. Despite meeting inclusion criteria, the studies varied substantially in diagnostic definitions and reference standards, which limits cross-study comparability and reduces confidence in biomarker generalisability.

**Table 1:** Main characteristics of selected studies in the systematic review.

First Author, Year	Sample Size	Age of Asthma Group (Mean ± SD)	Male Number (%)	Female Number (%)	Symptoms	Comorbidity	Sample Source	Evaluation Technique	Reference
Midyat et al., 2016	200	10.9 ± 3.3 years	103 (53.9%)	88 (46.1%)	Breathlessness, speech difficulty, alertness level, respiratory rate, accessory muscles, and wheezing	48 out of 95 asthmatic children (50.5%) were allergic/atopic.	Lymphocytes	Microarray	[31]
Beheshti et al., 2023	161	39 ± 1 weeks	73 (45%)	88 (55%)	Wheezing	NR	Saliva	RNA sequencing	[39]
Kang et al., 2021	103	9.26 ± 0.40 years	56 (54.37%)	47 (45.63%)	Bronchial asthma symptoms	NR	Serum	RT-qPCR	[40]
Elbehidy et al., 2016	175	9.0 ± 2.7 years	75 (42.9%)	100 (57.1%)	Wheezing	NR	Serum	RT-qPCR	[41]
Nakano et al., 2013	41	11 years	28 (68.3%)	13 (31.7%)	Recurrent wheezing, breathlessness, chest tightness, and coughing	NR	CD4^+^ T cells	RT-qPCR	[42]
Wang et al., 2023	195	6.3 ± 2.7 years	106 (54.4%)	89 (45.6%)	NR	NR	Mononuclear Cells (PBMCs)	RT-qPCR	[13]
Sawant et al., 2015	16	5 ± 0.2 years	NR	NR	Asthmatic children	In the asthma group, 25% subjects were atopic (IgE to egg/milk or other antigens)	Serum	RT-qPCR	[43]
Sharma et al., 2024	555	9.0 ± 1.8 years	326 (58.7%)	229 (41.3%)	Wheezing, coughing, and dyspnoea	NR	Serum	RNA sequencing	[30]
Zhou et al., 2021	51	<18 years	14 (48.3%)	15 (51.7%)	Paediatric patients with asthma	NR	Mononuclear cells (PBMCs)	RT-qPCR	[44]
Nasser et al., 2019	50	7.15 ± 4.24 years	35 (70%)	15 (30%)	Bronchial asthma with intermittent or persistent severity.	Allergic rhinitis was reported in 70% of the asthma group vs. 5% in controls (*p* = 0.001)	Serum	RT-qPCR	[45]
Liu et al., 2019	360	10.83 ± 3.06 years	199 (55.3%)	161 (44.7%)	Cough, wheezing, and dyspnoea	Allergy, rhinitis, and exposure to environmental tobacco smoke (ETS)	Serum	RT-qPCR	[46]
Tian et al., 2018	160	4.6 ± 1.1 years	72 (45%)	88 (55%)	Shortness of breath, audible expiratory wheezing	NR	Mononuclear cells (PBMCs)	RT-qPCR	[47]
Li et al., 2021	195	6.47 ± 2.7 years	106 (54.4%)	89 (45.6%)	Shortness of breath, cough, wheezing, and chest tightness	NR	Mononuclear cells (PBMCs)	RT-qPCR	[48]
Mendes et al., 2019	186	8.71 ± 0.84 years	91 (48.92%)	95 (51.08%)	Wheezing, dyspnoea, and dry cough	NR	Exhaled Breath Condensate (EBC)	RT-qPCR	[49]
Dong et al., 2018	200	6.50 ± 2.72 years	126 (63%)	74 (37%)	GINA	Food allergy	Whole blood	Microarray	[50]
Wang et al., 2015	54	6–13 years	29 (54%)	25 (46%)	GINA	NR	Plasma	Microarray	[37]
Zhang et al., 2018	33	3.8 ± 2.6 years	19 (57.6%)	14 (42.4%)	Acute asthma attacks	NR	Plasma	RT-qPCR	[36]
Rao et al., 2022	10	NR	NR	NR	Children with asthma exacerbation	NR	Mononuclear cells (PBMCs)	RNA sequencing	[51]
Chen et al., 2025	12	8.25 ± 3.17 years	7 (58.3%)	5 (41.7%)	NR	NR	CD4^+^ T cells	RT-qPCR	[52]
Dong et al., 2016	124	6.05 ± 2.15 years	78 (62.9%)	46 (37.1%)	Intermittent-to-mild asthma attacks	NR	Whole blood	Microarray	[53]
Yu et al., 2021	88	9 years	55 (62.5%)	33 (37.5%)	NR	NR	Serum	RT-qPCR	[54]
Mohany et al., 2024	158	10.5 ± 1.6 years	48 (30.4%)	110 (69.6%)	Wheezing, shortness of breath, and coughing	NR	Serum	RT-qPCR	[55]
Ibrahim et al., 2020	150	8.9 ± 1.3 years	53 (35.3%)	97 (64.7%)	GINA	Food allergy in 10% of asthmatic children	Serum	RT-qPCR	[56]
Bartel et al., 2020	27	11 ± 1.5 years	18 (66.7%)	9 (33.3%)	NR	NR	Nasal lavage fluids	RT-qPCR	[57]
Karam and Abd Elrahman, 2019	200	8.2 ± 3.4 years	NR	NR	Repeated wheezing and sharp breath	NR	Plasma	RT-qPCR	[32]
Tian et al., 2018	200	5.8 ± 1.9 years	94 (47%)	106 (53%)	NR	NR	Whole blood	RT-qPCR	[58]
Zhang et al., 2018	52	5.2 ± 3 years	33 (63.5%)	19 (36.5%)	GINA	History of other atopic diseases in 69.2% of children	CD14^+^ monocytes	Microarray	[38]
Zhang et al., 2016	60	10.86 ± 3.56 years	34 (56.7%)	26 (43.3%)	NR	NR	Plasma	RT-qPCR	[59]
He et al., 2022	170	4–11 years	106 (62.4%)	64 (37.6%)	NR	NR	Serum	RNA sequencing	[60]
Shi et al., 2019	30	15.87 ± 2.10 years	21 (70%)	9 (30%)	NR	NR	Bronchoalveolar lavage fluid	RT-qPCR	[61]
Eldosoky et al., 2023	76	8.4 ± 2.6 years	50 (62.5%)	30 (37.5%)	Wheezing, dyspnoea, and chronic cough	NR	Serum	RT-qPCR	[33]
Wang et al., 2023	332	269.52 ±11.33 days	161 (48.5%)	171 (51.5%)	Recurrent wheezing	NR	Cord blood	RNA sequencing	[62]
Liu et al., 2022	114	8.51 ± 3.65 years	71 (62.3%)	43 (37.7%)	NR	NR	Serum	RT-qPCR	[63]
Du et al., 2019	90	5.9 ± 2.1 years	34 (37.8%)	56 (62.2%)	NR	NR	Serum	RT-qPCR	[64]
Jiang et al., 2016	140	4.3 ±1.2 years	81 (57.9%)	59 (42.1%)	Recurrent wheezing (≥3 episodes)	Bronchitis	Plasma	RT-qPCR	[65]
Zhang et al., 2019	27	15.87 ± 2.10 months	21 (77.8%)	6 (22.2%)	NR	NR	Bronchoalveolar lavage fluid	RT-qPCR	[66]
Mirzakhani et al., 2025	389	38.67 ± 1.78 weeks	203 (52.2%)	184 (47.3%)	Wheezing	NR	Cord blood	RNA sequencing	[34]
Behairy et al., 2022	120	8.1 ± 2.3 years	66 (55%)	54 (45%)	Cough attacks, wheezy dyspnoea, chest tightness, expectoration, and tachypnoea	NR	Serum	RT-qPCR	[35]
Liu et al., 2012	12	5 ± 1 years	NR	NR	Wheezing episodes	NR	Lymphocytes	Microarray and RT-qPCR	[67]
Yin et al., 2019	76	6–12 years	42 (55.3%)	34 (44.7%)	NR	NR	Serum	RT-qPCR	[68]
Lu et al., 2023	183	10.1 years	94 (51.4%)	89 (48.6%)	NR	NR	Plasma	RT-qPCR	[69]
Dai et al., 2021	52	7.53 ± 2.16 years	35 (67.3%)	17 (32.7%)	NR	NR	Mononuclear cells (PBMCs)	RT-qPCR	[70]
Elnady et al., 2020	50	9.16 ± 1.78 years	35 (58.3%)	25 (41.7%)	NR	NR	Whole blood	RT-qPCR	[71]
Gu et al., 2022	39	5.33 ± 2.85 years	24 (61.5%)	15 (38.5%)	Wheezing and tachypnoea	NR	Mononuclear cells (PBMCs)	RT-qPCR	[72]
Kho et al., 2016	153	8.9 ± 2.0 years	89 (58%)	64 (42%)	Chronic asthma	NR	Serum	RT-qPCR	[29]
Zhang et al., 2022	30	6.13 ± 2.50 years	24 (66.67%)	12 (33.33%)	Wheezing	NR	Mononuclear cells (PBMCs)	RNA sequencing and RT-qPCR	[20]
Trifunovic et al., 2020	28	8.14 years	16 (57.1%)	12 (42.9%)	Acute wheezing and respiratory distress	Eczema	Nasal epithelial cells	Microarray	[21]

NR = not reported. GINA = Global Initiative for Asthma guidelines.

**Figure 3:**
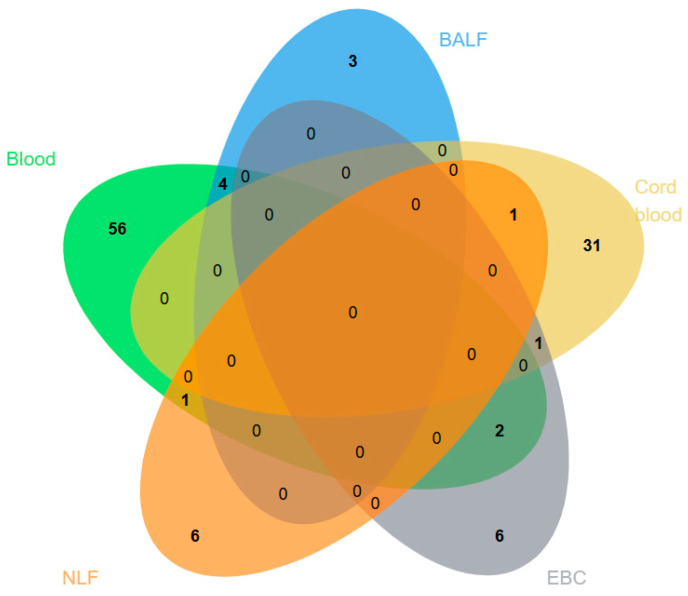
Overlap of significantly dysregulated microRNAs reported across the included childhood asthma studies by sample type. For profiling studies, miRNAs reported by the original authors as significantly dysregulated were included. For targeted RT-qPCR studies, only candidate miRNAs found to be significantly differentially expressed were included. miRNA nomenclature was harmonised before comparison to reduce duplication arising from naming differences. The figure summarises overlap among dysregulated miRNAs across studies and biological compartments and does not represent all miRNAs tested in the included studies.

After harmonisation of miRNA nomenclature, Figure 3 shows the overlap of unique significantly dysregulated miRNAs across sample types. These counts therefore refer to distinct miRNA entities rather than the total number of dysregulation events reported across studies. Among the 47 studies, 33 performed reverse transcription quantitative PCR (RT-qPCR), 8 used microarray analysis, and 6 employed RNA sequencing to quantify miRNA levels across various tissues and biofluids. Most studies (*n* = 38) assessed miRNA expression in blood or its components, including serum, plasma, lymphocytes, CD4^+^ T cells, and peripheral blood mononuclear cells (PBMCs). Cord blood miRNAs are reported separately from other blood samples because they reflect the perinatal immune environment and indicate asthma susceptibility and early-life risk biomarkers. While 3 studies targeted nasal lavage fluid (NLF), 3 focused on bronchoalveolar lavage fluid (BALF), 2 on cord blood, and 1 on exhaled breath condensate (EBC). The reviewed studies identified 70 significantly differentially expressed miRNAs in blood and its components, 8 in NLF, 8 in BALF, 39 in cord blood, and 9 in EBC. The distribution of these differentially expressed miRNAs across various sample types in asthmatic children compared to matched non-asthmatic healthy controls is indicated in Figure 3.

Twenty-one miRNAs (miR-21, miR-146a, miR-125b, miR-155, miR-34a, miR-221, miR-210, miR-22, miR-let-7a, miR-let-7c, miR-145-5p, miR-126, miR-495, miR-378, miR-125a-5p, miR-27b-3p, miR-143-3p, miR-98, miR-15a, miR-1248, and miR-200c-3p) were found in both blood and at least one airway-derived fluid (BALF, EBC, or NLF). Eight miRNAs (miR-21, miR-let-7a, miR-146a, miR-145-5p, miR-125a-5p, miR-378, miR-155, and miR-125b) were shared between blood and EBC. Six miRNAs (miR-21, miR-let-7a, miR-let-7b, miR-let-7c, miR-26a, and miR-146a) were shared between blood and BALF. Five miRNAs (miR-34a, miR-210, miR-125a-5p, miR-221, and miR-345) were shared between blood and NLF. Three miRNAs (miR-21, miR-let-7a, and miR-146a) were commonly detected across blood, BALF, and EBC, indicating airway-specific asthma responses. Two miRNAs (miR-145-5p and miR-125a-5p) were seen in cord blood and also in two airway fluids, suggesting a link to early-life asthma risk. The frequent overlap of miR-21 and miR-146a across different tissues supports the presence of a dispersed miRNA response in asthma rather than a response limited to localised airway response. Since these miRNAs circulate across diverse biofluids, reliable methods are needed to detect and differentiate closely related miRNA sequences in low abundance.

### 3.3. MicroRNAs for the Diagnosis of Childhood Asthma

Of the forty-seven included studies, only ten evaluated the diagnostic performance of miRNAs using receiver operating characteristic (ROC) curve analysis (Table 2). Because ROC analyses were reported in only a minority of studies, without pooled estimates or external validation cohorts, these findings should be interpreted as preliminary. Table 2 summarises miRNAs that were significantly differentially expressed between asthmatic or wheezing children and healthy controls, using a fold-change threshold of ≥2. Across these studies, twenty-four miRNAs were found to be significantly upregulated and eleven downregulated in asthmatic children compared to healthy controls (*p* < 0.05). Diagnostic performance was assessed using ROC analysis, with key scores including area under the curve (AUC), sensitivity, specificity, 95% confidence intervals (CIs), and statistical significance (*p* < 0.05). Based on this analysis, several miRNAs demonstrate potential as diagnostic biomarkers for distinguishing asthmatic children from healthy controls.

Among the miRNA candidates, miR-1 demonstrated the highest diagnostic accuracy, with an AUC of 0.977 (95% CI: 0.950–1.003, *p* = 0.001) in whole blood, indicating strong discriminatory power [58]. Similarly, miR-26a was significantly downregulated in serum and achieved an AUC of 0.96 (95% cut-off: 0.94), with a sensitivity of 83% and specificity of 93% (*p* < 0.002) [55]. Upregulated miR-210-3p, also measured in serum, yielded an AUC of 0.92 (95% CI: 0.85–0.994), sensitivity of 96%, and specificity of 68% (*p* < 0.001). miR-125b, also upregulated in serum, reached an AUC of 0.9, with 78% sensitivity and 84% specificity (*p* < 0.001). miR-126, upregulated in plasma, showed similarly strong diagnostic performance (AUC = 0.909, 95% CI: 0.831–0.987, *p* < 0.001).

Other miRNAs showed moderate diagnostic potential, including miR-494 (AUC = 0.821, *p* < 0.001); miR-21 (AUC = 0.800, sensitivity = 92.6%, specificity = 77.2%, *p* < 0.001), miR-196a2 (AUC = 0.800, *p* = 0.001); and miR-18a-5p (AUC = 0.799, sensitivity = 98.7%, specificity = 61.7%, *p* < 0.0001). Contrarily, miR-26a-5p measured in nasal lavage fluid showed a lower diagnostic value with an AUC of 0.604, indicating limited diagnostic performance as a standalone marker. Thus, miR-1, miR-26a, miR-210-3p, miR-126, and miR-125b may represent recurrent candidate markers for further validation, but the currently available evidence does not support their use as a validated diagnostic panel. (Table 2).

### 3.4. miRNAs Associated with Asthma Severity-Related Phenotypes in Children

Among the forty-seven enrolled studies, eleven particularly investigated the association between altered miRNA profiles and asthma phenotypes in children, compared to healthy controls. The findings have been summarised and stratified by asthma severity into mild, moderate, and severe asthma risk (Table 3). In lymphocytes, hsa-miR-193b was downregulated in children with mild asthma, whereas hsa-miR-497, let-7e, and miR-98 were upregulated in moderate to severe cases, indicating their potential utility in stratifying asthma severity [31]. In whole blood, miR-1 was consistently downregulated across all severity groups, suggesting an association with more severe asthma phenotypes relative to healthy controls, although direct evidence between-severity comparisons was limited [58]. In serum, miR-21 was upregulated across the severity spectrum from intermittent to severe asthma [40]. Other serum miRNAs, including miR-146a-5p, miR-210-3p, miR-378, and miR-196a2, were also consistently upregulated in mild, moderate, and severe asthma [33,56]. In contrast, miR-98-5p was downregulated across the same spectrum, highlighting its potential as a severity-related broad biomarker [64]. The circulating plasma miRNAs, miR-126 and miR-22, were significantly upregulated in children with severe asthma, while miR-21 was increased in mild and moderate cases [47,69]. Conversely, miR-26a was downregulated in moderate and severe asthma, suggesting its predictive value in more severe phenotypes [69].

### 3.5. Early-Life miRNAs as Predictors of Asthma Risk

Cord blood microRNA profiling revealed early-life expression levels that may serve as predictive biomarkers for subsequent asthma risk. miR-149-5p was upregulated in children who later developed persistent, transient, or new-onset asthma [34]. miR-99b-5p, miR-125a-5p, miR-200c-3p, and miR-145-5p were upregulated in cases of transient and new-onset asthma, indicating their role in early asthma [34]. In the nasal epithelium, miR-582-5p was upregulated in children with severe asthma, suggesting a localised airway response associated with disease severity [21]. Accordingly, miR-126, miR-21, miR-146a-5p, and miR-26a represent candidate biomarkers with preliminary evidence for severity assessment; however, cross-cohort replication and prospective validation are required before clinical use. The identified miRNAs showed varying expression patterns across different sample types. Only miR-21, miR-146a, and miR-let-7a were consistently detected across multiple tissue types, suggesting either true tissue-specific expression patterns or minimal technical variability across studies.

### 3.6. Retrieval of Asthma-Related Genes

A bioinformatic analysis was conducted on fifty-eight miRNAs exhibiting significant differential expression between asthmatic children and healthy controls (*p* < 0.05, fold change ≥ 1.5), of which thirty-one were upregulated, and twenty-seven were downregulated. We used the complete list of fifty-eight miRNAs for subsequent bioinformatics analyses. For diagnostic performance assessment (Table 2), we applied a threshold (fold change ≥ 2), resulting in a subset of thirty-five miRNAs. Using the miRWalk 3.0 database, integrating five prediction tools (miRBase v22.1, miRDB v6.0, TarPmiR, TargetScan v7.2, and miRTarBase v9.0) yielded 15,480 potential target genes. Cross-referencing these genes with the GeneCards database identified 56 unique genes clinically relevant to asthma, which were selected for further analysis.

**Figure 4:**
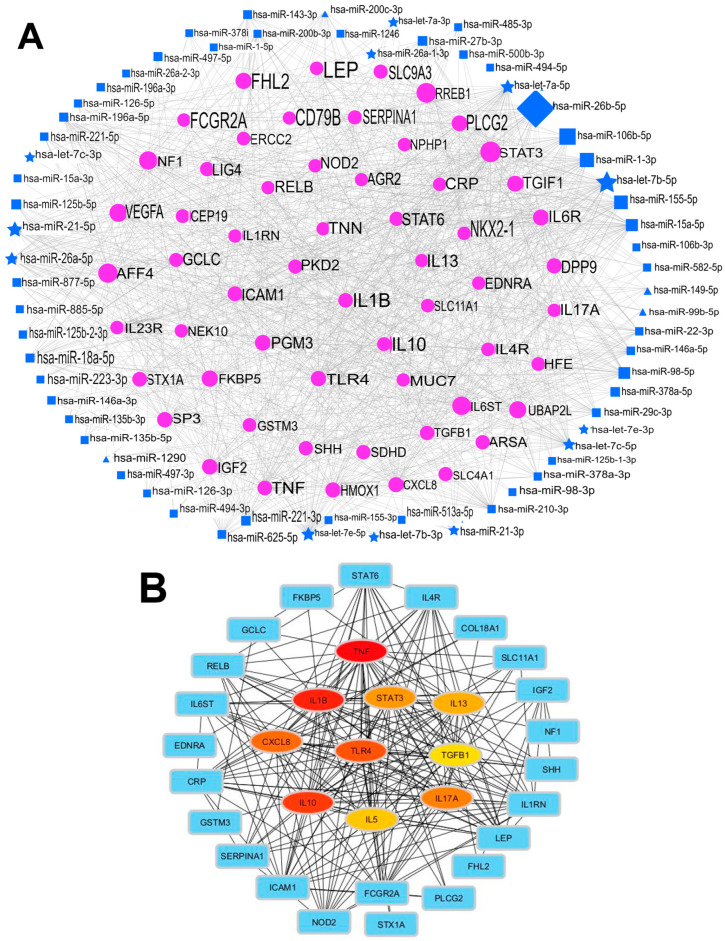
miRNA–gene interaction network associated with childhood asthma. (**A**) Regulatory network illustrating tissue-specific expression of miRNAs and their gene targets. Squares represent blood and its components (serum, plasma, lymphocytes, and CD14^+^ monocytes); triangles represent cord blood; diamonds represent nasal lavage fluid (NLF); stars represent bronchoalveolar lavage fluid (BALF); and circles represent exhaled breath condensate (EBC). Purple nodes indicate tissue-specific miRNAs. (**B**) Network of the top 10 hub genes identified from the protein–protein interaction analysis. The colour intensity correlates with the degree value, where darker colours indicate higher connectivity.

Functional annotation of these genes revealed enrichment in 265 Gene Ontology (GO) terms, including 733 biological processes, 6 cellular components, and 8 molecular functions, as well as 60 signalling pathways identified through KEGG pathway analysis. According to the *p*-value ranking, the top three biological processes identified were cell activation, leukocyte activation, and immune system process (Supplementary Figure S1A), suggesting potential molecular mechanisms underlying childhood asthma. KEGG pathway analysis revealed multiple signalling pathways enriched for asthma, with the top 10 pathways visualised in a bubble chart based on *p*-values and FDR correction (Supplementary Figure S1B). The asthma signalling pathway was ranked among the most significant, with a *p*-value of 0.00057, a strength of 1.72, a signal of 1.41, and a false discovery rate (FDR) of 0.000034. The distribution of miRNAs and their corresponding gene targets across different tissues and biofluids is illustrated in Figure 4.

### 3.7. Functional Analysis of Asthma-Related Targets

The 56 unique genes were subsequently mapped onto a protein–protein interaction (PPI) network using the STRING database, resulting in a network comprising 56 nodes and 234 edges (Supplementary Figure S2). The cytoHubba plugin in Cytoscape was then employed to identify key hub genes within this network. This analysis revealed 10 hub genes with the highest connectivity (Figure 4B). The top genes identified were TNF, IL1B, IL10, TLR4, CXCL8, IL17A, STAT3, IL13, IL5, and TGFB1, which achieved the highest degree of connectivity, suggesting a molecular network underlying childhood asthma. To assess the robustness of hub gene identification, a threshold-based sensitivity analysis was performed at 1.5 versus 2. TNF, IL5, IL13, TLR4, and IL1B consistently ranked among the top 10 hub genes under both thresholds, confirming the core hub gene stability. Minor rank shifts were observed among the remaining five hub genes (IL10, CXCL8, IL17A, STAT3, TGFB1).

### 3.8. Prediction of Drug Candidates for Childhood Asthma

**Figure 5:**
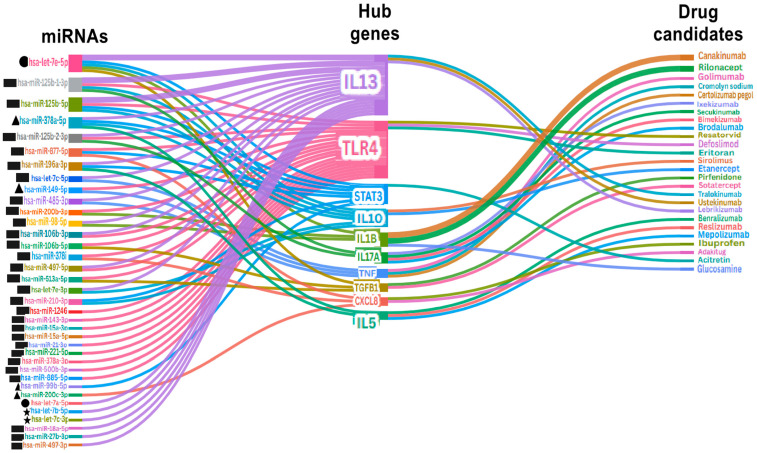
Exploratory network mapping showing the interaction between differentially expressed microRNAs, overlapped hub genes, and top-associated drugs. Squares represent blood and its components (serum, plasma, lymphocytes, CD14^+^ monocytes); triangles represent cord blood; diamonds represent nasal lavage fluid (NLF); stars represent bronchoalveolar lavage fluid (BALF); and circles represent exhaled breath condensate (EBC).

To illustrate the translational potential of the miRNAs identified in this systematic review, we performed an exploratory bioinformatics analysis to map these miRNAs onto hub gene networks and drug–gene interaction databases. This analysis highlighted several candidate therapeutic targets and repurposed drug classes. Ten hub genes with potential drug interactions were screened against the DSigDB library. Using DGIdb, a total of 339 drugs were identified as possible therapeutic candidates for treating childhood asthma. Applying a drug–gene interaction threshold ≥ 0.3 led to the identification of 62 drug candidates. Validation with the DrugBank database confirmed 26 candidate drugs from 62 DGIdb-derived compounds, verifying multiple drug–gene interactions, including approved asthma therapies such as mepolizumab, benralizumab, and lebrikizumab, providing biological plausibility for the network-derived targets, while not constituting independent therapeutic validation. Figure 5 illustrates the top-ranked potential therapeutic targets and their interactions with differentially expressed miRNAs and genes.

## 4. Discussion

We performed a systematic review to identify miRNA biomarkers associated with childhood asthma. Across the eligible studies, we catalogued a total of 128 candidate miRNAs, of which 35 demonstrated significant associations with childhood asthma. Several methodological limitations reduced comparability and interpretability. Many studies lacked critical clinical details, including comorbidities, concurrent treatments, sampling time points, and clearly defined phenotypes. Follow-up timing and symptom duration were reported inconsistently, and small sample sizes were frequently observed. Therefore, study quality and risk of bias were evaluated using the QUADAS-2 tool, which identified a few studies, particularly those conducted by [36,38], which were flagged as having a higher risk of bias, primarily due to their limited sample sizes (Figure 2). PRISMA-guided screening was used to ensure inclusion of studies comparing asthmatic phenotypes to healthy controls across sample types.

A major limitation of the evidence base is the substantial clinical and methodological heterogeneity across included studies. The study populations ranged from infancy to adolescence and combined preschool wheezing phenotypes with clinically diagnosed asthma, despite these representing distinct clinical entities with different prognostic implications. Similarly, cord blood miRNAs should be interpreted primarily as markers of future risk or susceptibility rather than diagnostic biomarkers of active childhood asthma. The included studies also analysed biologically distinct sample types, including circulating and airway-derived specimens, which are likely to reflect different pathobiological processes and therefore limit direct comparability. Moreover, important modifiers of miRNA expression, such as treatment exposure, disease severity, and coexisting atopy, were inconsistently reported, further constraining the interpretation of recurrent findings across studies.

A further limitation is that many included studies used candidate-based miRNA selection rather than unbiased profiling platforms. As a result, the miRNAs entered into the bioinformatic workflow were already enriched for molecules with suspected asthma relevance, introducing selection bias and increasing the likelihood of recovering canonical asthma-related pathways. Accordingly, the bioinformatic findings should be viewed as exploratory pathway contextualisation rather than unbiased target discovery.

Conflicting expression patterns across tissues present a further interpretive challenge. miR-21 and miR-146a showed consistent directional expression across tissues. However, miR-let-7a showed inconsistent expression direction between blood and BALF in one study, possibly reflecting tissue-specific biology or variation in detection thresholds. Such discrepancies should be taken into account when interpreting network-based regulatory predictions. Another key limitation is interpreting identified miRNAs as mechanistic drivers rather than secondary markers of inflammation or treatment exposure. Future longitudinal studies with standardised sampling, combined with experimental validation in airway epithelial models, are required to distinguish mechanistic roles from secondary biomarker effects.

Diagnosing asthma in young children remains challenging: objective tests (spirometry, airway inflammation measures) are often impractical in young children, and no single gold-standard diagnostic test exists. Additional technical challenges exist for conducting reliable tests, such as fractional exhaled nitric oxide (FeNO), which requires trained health professionals for accurate interpretation [73]. Furthermore, the heterogeneity of childhood asthma phenotypes (allergic vs. non-allergic, eosinophilic vs. neutrophilic) further complicates diagnosis [2,73]. These clinical and biological heterogeneities require phenotype-specific biomarker panels rather than universal markers. Of the 35 miRNAs achieving diagnostic threshold (fold change ≥ 2), a subset showed meaningful cross-cohort replication. miR-21 was the most consistently reported, upregulated in at least six independent cohorts across blood components [40,41,43,54,65,69]. miR-146a, miR-210-3p, and miR-146a-5p were each replicated in three or more studies with consistent expression directions [33,59,71]. miR-26a showed downregulation in serum and plasma, with consistent severity associations [55,61,69]. Conversely, miRNAs reported in single studies (e.g., miR-582-5p, miR-513a-5p) need independent replication before clinical use [21,53]. Current research increasingly combines respiratory function results with other risk factors such as diet, cold air exposure, smoke or dust inhalation, obesity, history of intensive care admission, poor inhaler technique, excessive bronchodilator use, and sputum or blood eosinophilia to improve diagnostic accuracy [10,74].

miRNAs emerged as promising biomarkers, offering specificity and sensitivity to support early diagnosis and severity risk assessment [75]. To our knowledge, this represents the first systematic review aimed at identifying expressed miRNA biomarkers in childhood asthma and assessing their potential as diagnostic and therapeutic indicators. Our paediatric focus reveals age-specific considerations not addressed in previous reviews covering all age groups [12,75]. A small subset of miRNAs showed recurrent differential expression across individual studies, but the evidence remains insufficient to define a clinically applicable diagnostic panel because of study heterogeneity, limited ROC reporting, and lack of external validation. (Table 3). Therefore, if these candidate miRNAs (Table 3) are to be evaluated in future clinical validation studies, integration with clinical records and relevant risk factors will be essential to enable accurate phenotyping and risk assessment. Translating these miRNA biomarkers into clinical practice presents several challenges. While miRNA measurement is potentially cost-effective compared to existing diagnostic methods, it demands specialised laboratory equipment and expertise [76]. Variations in sample collection, storage conditions, RNA extraction protocols, assay platforms, and data normalisation methods further contribute to miRNA measurement variability. Heterogeneity in biological sample types, including whole blood and its cellular components, bronchoalveolar lavage fluid, cord blood, exhaled breath condensate, and nasal lavage, presents variability challenges in miRNA profiling. This variability is exacerbated by the potential absence or significant dilution of miRNAs derived from airway epithelium in circulation, while immune cell-derived miRNAs may be detectable in blood but not in epithelial nasal samples. Furthermore, individual vulnerabilities and comorbidities increase the risk of misclassification bias in assessing asthma risk. Accurate diagnosis during phenotypic onset and between exacerbations ensures effective asthma management and prevents misdiagnosis or underestimating severity. Environmental triggers impacting susceptible children add further complexity to phenotype classification across studies [77]. Thus, standardising these factors improves the reliability and comparability of results across diagnostic facilities.

We complemented the systematic review with bioinformatic and tissue-stratified analyses to identify pathways and hub genes targeted by asthma miRNAs, as well as to prioritise candidate drugs. By grouping miRNA profiles according to sample type, we minimised cross-tissue variability and identified compartment-relevant targets: airway-derived miRNAs indicate potential inhaled or intranasal therapies, circulating miRNAs suggest systemically delivered biologics [78], while miRNA signals from cord blood may indicate early-life risk markers [34], supporting preventive interventions.

Network analysis identified hub genes as central nodes in biological processes linked to childhood asthma (Figure 4). Several regulate airway inflammation, immune cell activation, and tissue remodelling. For example, IL6, STAT3, and TNF control airway inflammation by promoting Th2/Th17 immune polarisation and triggering cytokine release, which sustains bronchial hyperresponsiveness [79].

TGFB1 and TLR4 contribute to airway remodelling, causing irreversible structural changes in severe asthma [80]. Their high connectivity within the network highlights their role as regulatory hubs integrating diverse signals from tissue-specific miRNAs. Their centrality marks them as key drivers of asthma pathogenesis, highlighting the importance of targeting their pathways in future therapies. The bioinformatic pipeline used here is confirmatory: it filters predicted miRNA targets against an asthma-specific gene database before enrichment analysis. This ensures that pathways and hub genes (TNF, IL5, IL13, TLR4, IL1B) reflect known asthma biology rather than novel drug discoveries. The analysis shows biological coherence between miRNA expression patterns from included studies and known asthma pathophysiology, supporting the disease relevance of the identified miRNA signatures.

The drug–gene interaction analysis (Figure 5) identifies several repurposable compounds that could modulate hub gene activity and reduce asthma-related pathways. Corticosteroids suppress IL6 and TNF expression through broad anti-inflammatory effects, while β2-agonists primarily limit airway remodelling [81]. Although Figure 5 illustrates that many drugs are not licensed for children, the overlap between predicted drug targets and key hub genes suggests therapeutic avenues that need further experimental validation. Monoclonal antibodies such as reslizumab, mepolizumab, and benralizumab have high interaction scores with asthma-associated genes ranging from 17.4 to 3.8, suggesting their potential in reducing asthma symptoms in children (Figure 5). Mepolizumab and benralizumab are approved biologics for paediatric patients aged 6 to 16 years with severe asthma. These therapies function by blocking active sites of the IL-5 receptor, which is regulated by the miR-let-7 family and is detectable in blood, exhaled breath condensate, and bronchoalveolar lavage fluid [82]. miRNA-based therapeutic interventions modulate translational control either by directly inhibiting the translation of target mRNAs or repressing protein production via mRNA degradation [83]. For instance, upregulation of the miR-let-7 family was confirmed in preschool wheezers (Table 2). This microRNA family activates the IL-13 pathway, thereby promoting the release of inflammatory cytokines [84]. Targeted therapies use biologic monoclonal antibodies to inhibit the IL-13 pathway, while corticosteroids and β2-agonists address inflammation and bronchoconstriction without directly affecting IL-13 [85]. Bioinformatics predictions require experimental validation and careful selection of administration routes because computational target prediction is limited by scarce licensing and safety data. These findings suggest that drug repurposing, with careful choice of administration routes, offers a promising strategy to improve personalised therapy and reduce asthma symptoms in children.

While numerous clinical validation studies have been conducted using adult populations, these findings have yet to be replicated in children’s cohorts. In children, several considerations are necessary before administering therapy across different care levels, from primary to specialist settings [86]. Asthma interventions currently focus on managing symptoms during acute wheezing episodes rather than molecular testing. After controlling symptoms, often with empirical treatments such as inhaled corticosteroids, objective diagnostic tests during stable conditions are important to assess asthma severity and guide treatment. A major concern is that therapy escalation can expose children to unnecessary risks, such as steroid side effects or antibiotic complications, without improving asthma control [87,88]. The lack of follow-up studies evaluating therapeutic responses contributes to current uncertainty in identifying differential responders. Therefore, further studies are needed to systematically compare the optimal dosing of these therapies across various administration routes, while accounting for the dynamic trajectories of asthma phenotypes and long-term follow-up outcomes. Advancing knockout miRNA-based therapeutic strategies that incorporate individual patient vulnerabilities is important for effectively targeting the molecular pathways driving childhood asthma risk.

Given the authors’ disclosed patent application related to miRNA-based asthma diagnosis, particular care was taken to present the findings conservatively; we emphasise that the available evidence is preliminary and does not support immediate clinical implementation.

## 5. Conclusions

In conclusion, the findings from this study indicate that miRNAs are promising candidate biomarkers for childhood asthma research, but current evidence is insufficient for near-term clinical implementation. miRNAs may offer new avenues for targeted and personalised therapeutic interventions. Yet, translating miRNA potentials into clinical practice demands the rigorous standardisation of sampling/assay pipelines, experimental validation, and integration with asthma risk factors.

## Figures and Tables

**Table 2:** Diagnostic performance of significantly differentially expressed miRNAs in asthmatic children across various tissues and biofluids.

miRNA	+/−	Sample	Expression (Fold Change)	Significance (*p*-Value *)	ROC Curve Analysis **	Reference
hsa-miR-497	+	Lymphocytes	>2	<0.05	Not reported	[31]
hsa-let-7e	+	Lymphocytes	>2	<0.05	Not reported	[31]
hsa-miR-98	+	Lymphocytes	>2	<0.05	Not reported	[31]
miR-26a-5p	+	Nasal lavage fluid (NLF)	2.5	=0.015	AUC: 0.604, 95% CI: not reported	[39]
miR-21	+	Serum	~3.5	<0.001	AUC: 0.80, 95% CI: 0.74–0.87, Sensitivity: 92.6%, Specificity: 77.2%, Cut-off value: >0.58, *p*-value: <0.001	[41]
miR-200b-3p	+	Serum	2.26	0.0001	Not reported	[30]
miR-885-5p	−	Serum	0.37	0.00012	Not reported	[30]
miR-15a	−	Serum	0.5	=0.03	Not reported	[45]
miR-155	+	Serum	2.32	<0.001	Not reported	[46]
miR-126	+	Plasma	3.6	<0.001	AUC = 0.909, 95% CI: 0.831–0.987, *p*-value < 0.001	[47]
miR-27b-3p	−	Whole blood	0.16	<0.01	Not reported	[50]
miR-Let-7c-5p	+	Plasma	2.72	<0.001	Not reported	[37]
miR-494	+	Plasma	2.15	<0.001	AUC = 0.821, 95% CI: not reported, *p*-value < 0.001	[37]
miR-22-3p	−	Whole blood	2.21	<0.05	Not reported	[53]
miR-513a-5p	−	Whole blood	2.07	<0.05	Not reported	[53]
miR-625-5p	−	Whole blood	2.17	<0.05	Not reported	[53]
miRNA-125b	+	Serum	4.27 ± 0.51	<0.001	AUC = 0.9, 95% CI: 1.2, sensitivity = 78%, specificity = 84%, *p*-value < 0.001	[55]
miRNA-26a	−	Serum	0.61 ± 0.08	<0.001	AUC = 0.96, 95% cut-off: 0.94, sensitivity = 83%, specificity = 93%, *p*-value < 0.002	[55]
miR-196a2	−	Serum	0.25	0.001	AUC = 0.8, 95% CI: 0.7–0.9, *p* = 0.001	[56]
miR-1	−	Whole blood	0.092	<0.05	AUC = 0.900, 95% CI: 0.857–0.943, *p* = 0.001) and diagnosing asthma severity: (AUC = 0.977, 95% CI: 0.950–1.003, *p* = 0.001)	[58]
miR-29c	−	CD14^+^ monocytes	0.35	<0.001	Not reported	[38]
miR-18a-5p	+	Serum	2.645	<0.0001	AUC = 0.799, (95% CI: 0.723–0.862, *p* < 0.0001), specificity 61.7%, sensitivity 98.7%	[60]
miR-210-3p	+	Serum	4.22	<0.001	AUC =0.92 (95% CI: 0.85–0.994, *p* < 0.001), the specificity is 68%, and the sensitivity is 96%	[33]
miR-98-5p	−	Serum	2.89 ± 0.09	<0.001	Not reported	[64]
miR-let-7a	+	Bronchoalveolar lavage fluid (BALF)	2.72 ± 0.48	<0.05	Not reported	[66]
miR-let-7b	+	Bronchoalveolar lavage fluid (BALF)	8.23 ± 1.64	<0.05	Not reported	[66]
miR-let-7c	+	Bronchoalveolar lavage fluid (BALF)	3.16 ± 0.62	<0.05	Not reported	[66]
miR-149-5p	+	Cord blood	2.87	<0.001	Not reported	[34]
miR-99b-5p	+	Cord blood	2.04	<0.001	Not reported	[34]
miR-200c-3p	+	Cord blood	2.08	=7.89 × 10^−9^	Not reported	[34]
miR-221	+	Lymphocytes	2	<0.05	Not reported	[67]
miR-485-3p	+	Lymphocytes	2.5	<0.05	Not reported	[67]
miR-146a	+	Whole blood	5.46	<0.05	Not reported	[71]
miR-106b	+	Whole blood	7.58	<0.05	Not reported	[71]
miR-582-5p	+	Nasal epithelial cells	24	<0.01	Not reported	[21]

+: upregulated. −: downregulated. ROC—receiver operating characteristic curve; AUC—area under the ROC curve; CI—95% confidence interval of the AUC; *p*-value—statistical significance of AUC (*p* < 0.05 considered significant). * *p*-values less than 0.05 were considered statistically significant. Diagnostic performance metrics are based on individual studies and may not be generalisable across different populations or technical platforms. ** ROC metrics are reported only where provided in the source studies. Several studies did not report confidence intervals or validation procedures.

**Table 3:** Differentially expressed miRNAs associated with childhood asthma severity across various tissues and biofluids.

miRNA	+/−	Sample	Severity of the Asthma Group Versus the Healthy Control	Reference
hsa-miR-193b	−	Lymphocytes	mild	[31]
hsa-miR-497	+	Lymphocytes	moderate/severe	[31]
hsa-let-7e	+	Lymphocytes	moderate/severe	[31]
hsa-miR-98	+	Lymphocytes	moderate/severe	[31]
miR-21	+	Serum	intermittent, mild, moderate, and severe	[40]
miR-126	+	Plasma	severe	[47]
miR-196a2	+	Serum	severe	[56]
miR-1	−	Whole blood	mild, moderate, and severe	[58]
miR-146a-5p	+	Serum	mild, moderate, and severe	[33]
miR-210-3p	+	Serum	mild, moderate, and severe	[33]
miR-98-5p	−	Serum	mild, moderate, and severe	[64]
miR-149-5p	+	Cord blood	persistent, transient, and new-onset	[34]
miR-99b-5p	+	Cord blood	transient and new-onset	[34]
miR-125a-5p	+	Cord blood	transient and new-onset	[34]
miR-200c-3p	+	Cord blood	transient and new-onset	[34]
miR-145-5p	+	Cord blood	Transient and new-onset	[34]
miR-378	+	Serum	mild, moderate, and severe	[35]
miR-21	+	Plasma	mild and moderate	[69]
miR-22	+	Plasma	severe	[69]
miR-26a	−	Plasma	moderate and severe	[69]
miR-582-5p	+	Nasal epithelial cells	severe	[21]

+: upregulated. −: downregulated.

## Data Availability

The original contributions presented in this study are included in the article/Supplementary Material. Further inquiries can be directed to the corresponding author.

## Supplementary Materials

The following supporting information can be downloaded at https://www.mdpi.com/article/10.3390/jpm16040179/s1: Figure S1: Bubble plots showing the significantly enriched biological processes (A) and KEGG pathways (B); Figure S2: Protein-protein interaction (PPI) network of differentially expressed genes associated with asthma; Table S1: Search strategy and results; Table S2: Prisma 2020 checklist.
