# Supplementary material for: MicroRNAs as Diagnostic and Therapeutic Biomarkers in Childhood Asthma: A Systematic Review with Bioinformatics Analysis
Source: J Pers Med. 2026 Mar 25;16(4):179. doi: 10.3390/jpm16040179 (PMC13117330; doi:10.3390/jpm16040179)
Supplement: Supplementary file 1 [file jpm-16-00179-s001.zip › jpm-4186825-supplementary.pdf]

Supplementary Table S1. Search strategy and results.

Search date: 19/03/2025

Database: MEDLINE (Ovid)

Step	Keywords	Results
1	asthma$.af.	241795
2	asthma$.ab,ct,kw,ti.	216464
3	wheez$.ab,ct,kw,ti.	16963
4	respir$.ab,ct,kw,ti.	913696
5	1 or 2 or 3 or 4	1095549
6	preschool$.ab,ct,kw,ti.	40144
7	child$.ab,ct,kw,ti.	2940231
8	offspring$.ab,ct,kw,ti.	98585
9	school$.ab,ct,kw,ti.	423158
10	young$.ab,ct,kw,ti.	1924855
11	6 or 7 or 8 or 9 or 10	4682656
12	non-coding RNA$.ab,ct,kw,ti.	48717
13	small RNA$.ab,ct,kw,ti.	19078
14	microRNA$.ab,ct,kw,ti.	182464
15	miRNA$.ab,ct,kw,ti.	115004
16	circulating microRNA$.ab,ct,kw,ti.	4241
17	circulating miRNA$.ab,ct,kw,ti.	3593
18	exosomal miRNA$.ab,ct,kw,ti.	2181
19	serum miRNA$.ab,ct,kw,ti.	1254
20	plasma miRNA$.ab,ct,kw,ti.	978
21	12 or 13 or 14 or 15 or 16 or 17 or 18 or 19 or 20	233382
22	5 and 11 and 21	403

Database: Embase (Ovid)

Step	Keywords	Results
1	asthma$.af.	434407
2	asthma$.ab,ct,kw,ti.	392525
3	wheez$.ab,ct,kw,ti.	46724
4	respir$.ab,ct,kw,ti.	1441243
5	1 or 2 or 3 or 4	1765208
6	preschool$.ab,ct,kw,ti.	769725
7	child$.ab,ct,kw,ti.	3791859
8	offspring$.ab,ct,kw,ti.	124357
9	school$.ab,ct,kw,ti.	959560
10	young$.ab,ct,kw,ti.	1837019
11	6 or 7 or 8 or 9 or 10	5638983
12	non-coding RNA$.ab,ct,kw,ti.	58922
13	small RNA$.ab,ct,kw,ti.	23006
14	microRNA$.ab,ct,kw,ti.	277937
15	miRNA$.ab,ct,kw,ti.	148330
16	circulating microRNA$.ab,ct,kw,ti.	6233
17	circulating miRNA$.ab,ct,kw,ti.	5346

18	exosomal miRNA$.ab,ct,kw,ti.	2977
19	serum miRNA$.ab,ct,kw,ti.	1998
20	plasma miRNA$.ab,ct,kw,ti.	1567
21	12 or 13 or 14 or 15 or 16 or 17 or 18 or 19 or 20	322281
22	5 and 11 and 21	504

Database: E-Journals (Ovid)

Step	Keywords	Results
1	asthma$.af.	391925
2	asthma$.ab,ct,kw,ti.	371627
3	wheez$.ab,ct,kw,ti.	37341
4	respir$.ab,ct,kw,ti.	1245111
5	1 or 2 or 3 or 4	1571254
6	preschool$.ab,ct,kw,ti.	64932
7	child$.ab,ct,kw,ti.	3245794
8	offspring$.ab,ct,kw,ti.	149208
9	school$.ab,ct,kw,ti.	656401
10	young$.ab,ct,kw,ti.	1597443
11	6 or 7 or 8 or 9 or 10	4953756
12	non-coding RNA$.ab,ct,kw,ti.	67181
13	small RNA$.ab,ct,kw,ti.	25366
14	microRNA$.ab,ct,kw,ti.	194669
15	miRNA$.ab,ct,kw,ti.	165702
16	circulating microRNA$.ab,ct,kw,ti.	5641
17	circulating miRNA$.ab,ct,kw,ti.	5957
18	exosomal miRNA$.ab,ct,kw,ti.	3296
19	serum miRNA$.ab,ct,kw,ti.	2266
20	plasma miRNA$.ab,ct,kw,ti.	1772
21	12 or 13 or 14 or 15 or 16 or 17 or 18 or 19 or 20	302534
22	5 and 11 and 21	178

Database: CINAHL (EBSCOhost)

Step	Keywords	Results
1	TI asthma* OR AB asthma*	18,662
2	TI child* OR AB child*	341,439
3	(MM "MicroRNAs+") OR (MH "Circulating MicroRNA") OR "microRNA"	3,245
4	(MH "Respiratory Syncytial Virus, Human") OR (MH "Respiratory Sounds+") OR (MH "Asthma+")	17,641
5	TX preschool*	172,533
6	TX wheez*	5,827
7	TX child*	985,690
8	TX non-coding RNA* OR TX small RNA* OR TX microRNA* OR TX miRNA* OR TX circulating microRNA* OR TX exosomal miRNA* OR TX serum	19,029

	miRNA* OR plasma miRNA*	
9	(MH "Asthma+") OR "Asthma" OR (MH "Asthma, Occupational") OR (MH "Asthma, Aspirin-Induced") OR (MH "Asthma, Exercise-Induced") OR (MH "Cough-Variant Asthma") OR (MH "Asthma-Chronic Obstructive Pulmonary Disease Overlap Syndrome") OR (MH "Dyspnea, Paroxysmal")	19,913
10	S1 OR S9	20,089
11	S4 OR S6	20,768
12	S2 OR S5 OR S7	985,951
13	S3 OR S8	19,029
14	S10 AND S11 AND S12 AND S13	62

Database: E-Journal (EBSCOhost)

Step	Keywords	Results
1	TI asthma* OR AB asthma*	120,488
2	TI child* OR AB child*	1,516,482
3	(MM "MicroRNAs+") OR (MH "Circulating MicroRNA") OR "microRNA"	37,080
4	(MH "Respiratory Syncytial Virus, Human") OR (MH "Respiratory Sounds+") OR (MH "Asthma+")	0
5	TX preschool*	43,730
6	TX wheez*	9,274
7	TX child*	1,705,842
8	TX non-coding RNA* OR TX small RNA* OR TX microRNA* OR TX miRNA* OR TX circulating microRNA* OR TX exosomal miRNA* OR TX serum miRNA* OR plasma miRNA*	209,047
9	(MH "Asthma+") OR "Asthma" OR (MH "Asthma, Occupational") OR (MH "Asthma, Aspirin-Induced") OR (MH "Asthma, Exercise-Induced") OR (MH "Cough-Variant Asthma") OR (MH "Asthma-Chronic Obstructive Pulmonary Disease Overlap Syndrome") OR (MH "Dyspnea, Paroxysmal")	126,714
10	S1 OR S9	134,367
11	S4 OR S6	9,274
12	S2 OR S5 OR S7	1,710,031
13	S3 OR S8	209,047
14	S10 AND S11 AND S12	3,294
15	S3 OR S15	40,374
16	S2 AND S6 AND S13	4

Database: Medline (EBSCOhost)

Step	Keywords	Results

1	TI asthma* OR AB asthma*	133,999
2	TI child* OR AB child*	1,305,710
3	(MM "MicroRNAs+") OR (MH "Circulating MicroRNA") OR "microRNA"	104,481
4	(MH "Respiratory Syncytial Virus, Human") OR (MH "Respiratory Sounds+") OR (MH "Asthma+")	128,390
5	TX preschool*	868,269
6	TX wheez*	15,049
7	TX child*	2,882,537
8	TX non-coding RNA* OR TX small RNA* OR TX microRNA* OR TX miRNA* OR TX circulating microRNA* OR TX exosomal miRNA* OR TX serum miRNA* OR plasma miRNA*	277,868
9	(MH "Asthma+") OR "Asthma" OR (MH "Asthma, Occupational") OR (MH "Asthma, Aspirin-Induced") OR (MH "Asthma, Exercise-Induced") OR (MH "Cough-Variant Asthma") OR (MH "Asthma-Chronic Obstructive Pulmonary Disease Overlap Syndrome") OR (MH "Dyspnea, Paroxysmal")	163,313
10	S1 OR S9	165,223
11	S4 OR S6	133,460
12	S2 OR S5 OR S7	2,883,059
13	S3 OR S8	277,868
14	S10 AND S11 AND S12	401

Database: Web of Science Core Collection

Step	Keywords	Results
1	ALL=(asthma* OR wheez* OR respirat* OR cough*)	1,378,480
2	ALL=(preschool* OR school* OR child* OR offspring* OR young*)	7,367,860
3	ALL=("non-coding RNA*" OR "small RNA*" OR "microRNA*" OR "miRNA*" OR "circulating microRNA*" OR "circulating miRNA*" OR "exosomal miRNA*" OR "serum miRNA*" OR "plasma miRNA*")	260,771
4	#5 AND #4 AND #3	1,096

Database: Scopus

Step	Keywords	Results
1	"asthma" AND "wheezing" AND respirat cough preschool school "child" "children" offspring young AND non-coding RNA AND "circulating miRNA" small RNA AND "microRNA" AND "miRNA" OR circulating microRNA OR exosomal miRNA serum miRNA plasma miRNA	30,136

Database: PubMed

Step	Keywords	Results
1	((asthma*[Title/Abstract] OR wheez*[Title/Abstract] OR respirat*[Title/Abstract] OR cough*[Title/Abstract]) AND (preschool*[Title/Abstract] OR school*[Title/Abstract] OR child*[Title/Abstract] OR offspring*[Title/Abstract] OR young*[Title/Abstract])) AND ("non-coding RNA*"[Title/Abstract] OR "small RNA*"[Title/Abstract] OR "microRNA*"[Title/Abstract] OR "miRNA*"[Title/Abstract] OR "circulating microRNA*"[Title/Abstract] OR "circulating miRNA*"[Title/Abstract] OR "exosomal miRNA*"[Title/Abstract] OR "serum miRNA*"[Title/Abstract] OR "plasma miRNA*"[Title/Abstract])	518

Database: ProQuest

Step	Keywords	Results
1	noft(asthma* OR wheez* OR "Respirat* Sound*" OR Cough*) AND noft(offspring* OR child* OR preschool*) AND noft("MicroRNA*" OR "Circulating MicroRNA*" OR "microRNA*" OR non-coding RNA* OR TX small RNA* OR TX microRNA* OR TX miRNA* OR TX circulating microRNA* OR TX exosomal miRNA* OR TX serum miRNA* OR plasma miRNA*) AND stype.exact("Dissertations & Theses")	10

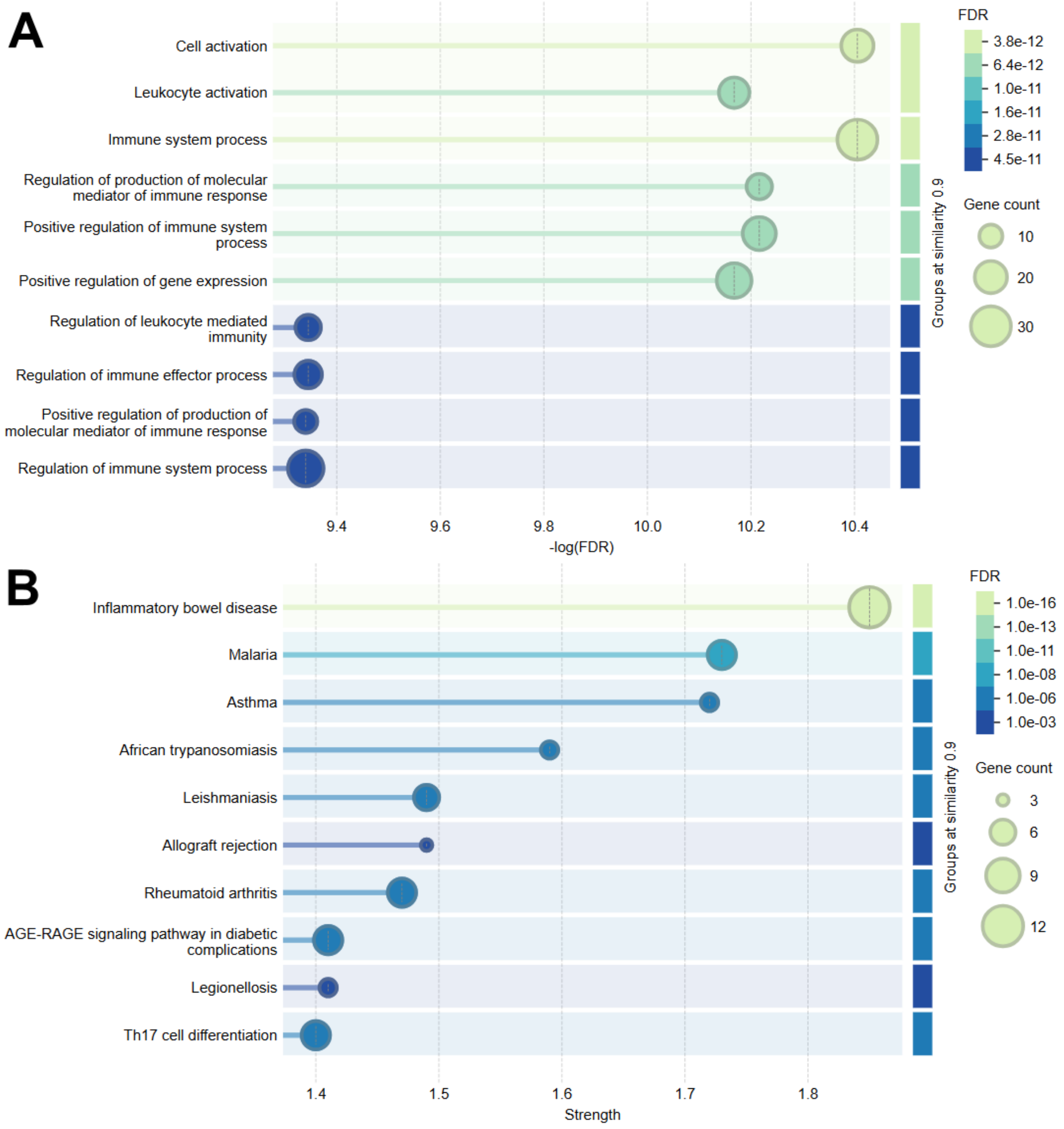

Supplementary Figure S1. Bubble plots showing the significantly enriched biological processes (A) and KEGG pathways (B). Y-axis displays the terms of enriched processes and pathways, and the x-axis indicates the types of molecular sets subjected to enrichment analysis. The dot size corresponds to the overlap ratio, which is calculated by dividing the proportion of overlap (number of molecules overlapped/total number of genes in the term) for the set size. The dots are coloured by the -log10 adjusted p-values, spanning from blue (larger p-value) to red (smaller p-value). The terms on the y-axis are grouped according to their GO and KEGG categories, which are annotated by colored bars.

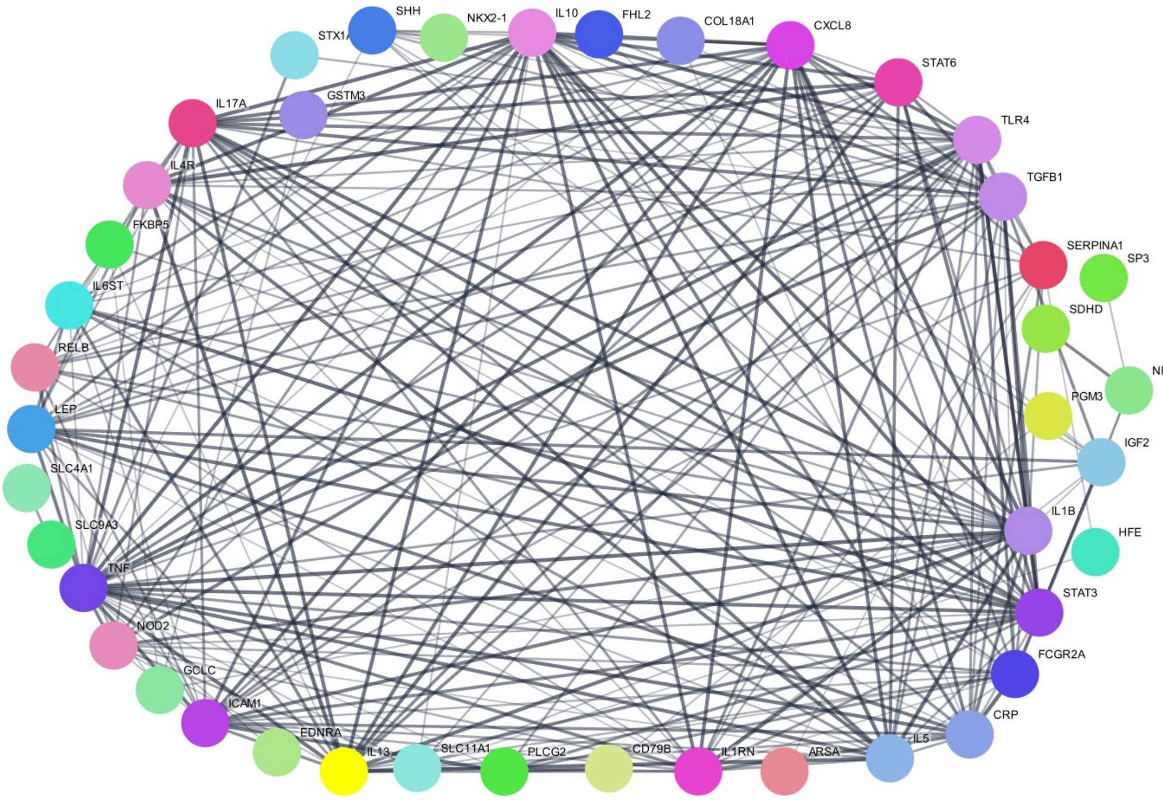

Supplementary Figure S2. **Protein-protein interaction (PPI) network of differentially expressed genes associated with asthma**. Nodes represent proteins, while edges indicate protein–protein associations. Light blue and purple edges denote known interactions, whereas dark blue, green, and red edges indicate predicted interactions.

Supplementary Table S2. Prisma 2020 checklist.

Section and Topic	Item #	Checklist item	Location where item is reported
**TITLE**			
Title	1	Identify the report as a systematic review.	Title: "MicroRNAs as Diagnostic and Therapeutic Biomarkers in Childhood Asthma: A Systematic Review with Bioinformatics Analysis ". Page1
**ABSTRACT**			
Abstract	2	See the PRISMA 2020 for Abstracts checklist.	Includes Background, Objective, Methods, Results, and Conclusion. Page1 (Abstract).
**INTRODUCTION**			
Rationale	3	Describe the rationale for the review in the context of existing knowledge.	Discusses diagnostic challenges in preschoolers, lack of gold-standard tests, and the potential of miRNAs as stable biomarkers in childhood asthma. Pages 2-3 (Introduction)
Objectives	4	Provide an explicit statement of the objective(s) or question(s) the review addresses.	To identify miRNAs differentially expressed in preschool wheezing/childhood asthma, assess severity risk, and explore precision-therapy relevance. Page 1 (Objective in Abstract)
**METHODS**			
Eligibility criteria	5	Specify the inclusion and exclusion criteria for the review and how studies were grouped for the syntheses.	Included English full-text quantitative studies in children <18 years that compared wheeze/asthma groups with healthy controls and reported differentially expressed miRNAs. Required clear/robust asthma diagnosis criteria for moderate-to-severe asthma based on at least one internationally recognised guideline (BTS, GINA, GEMA, GDPBAP, ATS/ERS). Also included studies reporting fetal cord-blood miRNAs associated with future asthma. Excluded: unpublished theses and master's dissertations; reviews, clinical trials, editorials/opinion overviews, conference abstracts and government reports; studies reporting no statistically significant miRNA differences; studies without a healthy control comparison; in vitro-only or animal-only studies; and studies focused exclusively on other genetic respiratory conditions or non-asthma causes of respiratory disease. Pages 3-4 (Methods - Eligibility criteria).
Information sources	6	Specify all databases, registers, websites, organisations, reference lists and other sources searched or consulted to identify studies. Specify the date when each source was last searched or consulted.	Sources searched: MEDLINE, Embase, CINAHL, PubMed, Scopus, Ovid, ProQuest thesis, E-journals, Web of Science. Coverage: Jan 1, 2011 to Mar 19, 2025; last search date Mar 19, 2025. Page 3 (Methods - Search Strategy) and page 6 (Results - Characteristics).

Section and Topic	Item #	Checklist item	Location where item is reported
Search strategy	7	Present the full search strategies for all databases, registers and websites, including any filters and limits used.	Search used Boolean strings with synonyms and MeSH terms. Full database-specific strategies are stated to be in Supplementary Table S1.
Selection process	8	Specify the methods used to decide whether a study met the inclusion criteria of the review, including how many reviewers screened each record and each report retrieved, whether they worked independently, and if applicable, details of automation tools used in the process.	Three-step screening (title, abstract, full text). First reviewer (AIA) screened; other reviewers (MAK, JJ, IO, EV) independently repeated screening; discrepancies resolved by discussion. Page 3 (Methods - Study Selection).
Data collection process	9	Specify the methods used to collect data from reports, including how many reviewers collected data from each report, whether they worked independently, any processes for obtaining or confirming data from study investigators, and if applicable, details of automation tools used in the process.	Full texts screened and data manually extracted using predefined criteria; duplicates removed. AIA and MAK independently conducted the data extraction. Page 4 (Methods - Data extraction).
Data items	10a	List and define all outcomes for which data were sought. Specify whether all results that were compatible with each outcome domain in each study were sought (e.g. for all measures, time points, analyses), and if not, the methods used to decide which results to collect.	Differentially expressed miRNAs, (up/down regulations; expression levels; fold change; p-values) and, where available, diagnostic performance (ROC/AUC, sensitivity, specificity, 95% CI). Severity/phenotype- and early-life prediction-related miRNAs were also extracted. Page 4 (Methods- Data extraction).
	10b	List and define all other variables for which data were sought (e.g. participant and intervention characteristics, funding sources). Describe any assumptions made about any missing or unclear information.	Variables included study details (title/authors/year/location), participant characteristics (sample size, age group, sex distribution), clinical data (phenotypes, setting, symptoms, comorbidities), sample source, and miRNA measurement method. Page 4 (Methods - Data extraction) and Table 1 (pages 8-12).
Study risk of bias assessment	11	Specify the methods used to assess risk of bias in the included studies, including details of the tool(s) used, how many reviewers assessed each study and whether they worked independently, and if applicable, details of automation tools used in the process.	Risk of bias assessed using QUADAS-2 by two independent reviewers; rated as low/high/some concerns (guidance accessed 23 Jun 2025). Page 4 (Methods - Quality assessment and risk of bias).
Effect measures	12	Specify for each outcome the effect measure(s) (e.g. risk ratio, mean difference) used in the synthesis or presentation of results.	Results presented when fold change (≥1.5) and p-value (<0.05) for differential expression and as diagnostic accuracy metrics (AUC, sensitivity, specificity, 95% CI when reported). Page 4 (Methods- Data extraction) and Table 2 (pages 13-15)
Synthesis methods	13a	Describe the processes used to decide which studies were eligible for each synthesis (e.g. tabulating the study intervention characteristics and comparing against the planned groups for each synthesis (item #5)).	Studies eligible for synthesis if they met eligibility criteria and reported significant differential miRNA expression vs controls (p<0.05; fold change >=1.5). Syntheses were organised by clinical question (diagnosis, severity/phenotype, early-life prediction) and by tissue/biofluid. Methods (pages 3-4) and Results subsections (pages 6-17).
	13b	Describe any methods required to prepare the data for presentation or synthesis, such as handling of missing summary statistics, or data conversions.	Applied thresholds (p<0.05; fold change >=1.5). Target prediction with miRWalk 3.0 (>90% confidence; 5' arm only; targets supported by >=3 databases). Asthma gene validation with GeneCards. Enrichment with

Section and Topic	Item #	Checklist item	Location where item is reported
			DAVID v6.8 (GO/KEGG; p<0.05). PPI with STRING (min score 0.4). Network visualisation with miRNet and Cytoscape (cytoHubba). Drug-gene interactions with DGIdb (score >=0.3) and validation with DrugBank. Pages 4-5 (Methods - Bioinformatics).
	13c	**Describe any methods used to tabulate or visually display results of individual studies and syntheses.**	Flow diagram (Fig1, page 6); QUADAS-2 assessments (Fig2, page 7); miRNA overlap (Fig3, page 12); miRNA-gene network (Fig4, page 17); drugs network (Fig5 p24). Study characteristics (Table 1, pages 7-12); diagnostic performance (Table 2, pages 14-15); severity miRNAs (Table 3, pages 15-16). miRNA interactions (Fig5, page 18).
	13d	Describe any methods used to synthesize results and provide a rationale for the choice(s). If meta-analysis was performed, describe the model(s), method(s) to identify the presence and extent of statistical heterogeneity, and software package(s) used.	Narrative synthesis with systematic comparison across studies; meta-analysis not performed due to heterogeneity. Bioinformatic/network analyses described in Methods (pages 4-6).
	13e	Describe any methods used to explore possible causes of heterogeneity among study results (e.g. subgroup analysis, meta-regression).	No formal heterogeneity analyses (e.g., subgroup meta-analysis/meta-regression). Qualitative exploration of miRNAs was done using sample source, detection method and phenotype categories. Results (pages 6-16).
	13f	Describe any sensitivity analyses conducted to assess robustness of the synthesized results.	No sensitivity analyses conducted.
Reporting bias assessment	14	Describe any methods used to assess risk of bias due to missing results in a synthesis (arising from reporting biases).	No formal reporting-bias assessment reported. Potential biases discussed. English-language restriction (page 3) and attempt to reduce publication bias by screening ProQuest dissertations (page 6).
Certainty assessment	15	Describe any methods used to assess certainty (or confidence) in the body of evidence for an outcome.	Study design limitations assessed via QUADAS-2 (GRADE not explicitly used). Methods - Quality assessment (page 4).
**RESULTS**			
Study selection	16a	Describe the results of the search and selection process, from the number of records identified in the search to the number of studies included in the review, ideally using a flow diagram.	3,873 records identified; 2,121 after duplicates; 1,934 excluded at title/abstract; 185 full texts assessed; 47 studies included. Additionally, 10 ProQuest dissertations screened (none eligible). Results - Characteristics (pages 6-7) and Fig1 (page 6).
	16b	Cite studies that might appear to meet the inclusion criteria, but which were excluded, and explain why they were excluded.	Reasons for exclusion at screening included animal/in vitro; wrong setting/outcomes, and preprint. Figure 1: PRISMA flow diagram (page 6).
Study characteristics	17	Cite each included study and present its characteristics.	Table 1: Main characteristics of selected studies (first author, year, sample size, age, symptoms, etc.). Pages (7-12).
Risk of bias in studies	18	Present assessments of risk of bias for each included study.	Results - Quality assessment: 42 high quality, 3 fair, 2 low quality; summarised in Fig2 (Page 7).

Section and Topic	Item #	Checklist item	Location where item is reported
Results of individual studies	19	For all outcomes, present, for each study: (a) summary statistics for each group (where appropriate) and (b) an effect estimate and its precision (e.g. confidence/credible interval), ideally using structured tables or plots.	Cross-tissue miRNA profiles summarised (direction of change, fold change/p-values when reported) and diagnostic metrics with precision where available (e.g., 95% CI in Table 2). Not all studies reported complete group summary statistics. Tables 1-3 (pages 7-16) and Results text (pages15-21).
Results of syntheses	20a	For each synthesis, briefly summarise the characteristics and risk of bias among contributing studies.	Synthesis narrative describes contributing studies by tissue/biofluid and clinical phenotype; overall risk of bias summarised. pp15-21 Results subsections (pages 6-18) and QUADAS-2 risk assessment (page 7).
	20b	Present results of all statistical syntheses conducted. If meta-analysis was done, present for each the summary estimate and its precision (e.g. confidence/credible interval) and measures of statistical heterogeneity. If comparing groups, describe the direction of the effect.	47 included studies identified 58 differentially expressed miRNAs (31 upregulated, 27 downregulated), with recurrent miRNAs across blood, nasal samples, bronchoalveolar lavage fluid (BALF) and exhaled breath condensate (pp15-17; Fig3, page 12). Diagnostic-performance results were summarised from individual studies (Table 2, pages 14-15), including AUC, sensitivity, specificity and 95% CI when reported. Bioinformatic synthesis mapped the 58 miRNAs to asthma-related targets and pathways (Figs 4-5): 56 unique asthma-related genes were retained for enrichment, 10 hub genes were highlighted (including TNF, IL5, IL13, TLR4), and drug-gene interaction screening identified 339 candidate drugs; applying a DGIdb interaction score >=0.3 yielded 62 candidates, with DrugBank validation confirming 26 drugs (including mepolizumab, benralizumab and lebrikizumab) (Fig5, page 18). No meta-analysis/statistical pooling was conducted (heterogeneity precluded meta-analysis; page 4-6). Key narrative findings are reported in Results (pages 6-16) and Abstract (page 1).
	20c	Present results of all investigations of possible causes of heterogeneity among study results.	58 differentially expressed miRNAs (31 up, 27 down); Functional enrichment of 10 hub genes and drug candidate validation. Results (pages 13-18).
	20d	Present results of all sensitivity analyses conducted to assess the robustness of the synthesized results.	No sensitivity analyses conducted.
Reporting biases	21	Present assessments of risk of bias due to missing results (arising from reporting biases) for each synthesis assessed.	Missing results were reported across included studies. Narrative comments on bias were included. Table 2 (pages 14-15) and Discussion (page 19).
Certainty of evidence	22	Present assessments of certainty (or confidence) in the body of evidence for each outcome assessed.	High proportion of high-quality studies supports miRNAs as reliable biomarkers for diagnosis, deep phenotyping, and severity monitoring. Results - Quality assessment (Page 7).
**DISCUSSION**			

Section and Topic	Item #	Checklist item	Location where item is reported
Discussion	23a	Provide a general interpretation of the results in the context of other evidence.	Interprets findings in context of asthma diagnosis challenges and prior biomarker evidence; discusses biological pathways, hub genes and potential therapies. Discussion (pages 18-20).
	23b	Discuss any limitations of the evidence included in the review.	Limitations included (variable diagnostic criteria; patient-selection/reference-standard bias; missing clinical details such as comorbidities/treatments/time points; inconsistent follow-up; small sample sizes). Discussion (pages 18-20).
	23c	Discuss any limitations of the review processes used.	English-language restriction acknowledged as potential bias (page 3); heterogeneity preventing meta-analysis (page 4 and page 6); grey literature search yielded no eligible dissertations (page 6).
	23d	Discuss implications of the results for practice, policy, and future research.	Implications for using miRNA panels for diagnosis/phenotyping/severity monitoring and for identifying therapeutic targets. Bioinformatic results highlight targetable pathways and potential therapies. Discussion (pages 18-20) and Conclusion (page 21).
**OTHER INFORMATION**			
Registration and protocol	24a	Provide registration information for the review, including register name and registration number, or state that the review was not registered.	Registered in PROSPERO (CRD420250655715). Methods (page 3).
	24b	Indicate where the review protocol can be accessed, or state that a protocol was not prepared.	Protocol submitted to PROSPERO; protocol access details/link not provided in the manuscript. Methods (page 3).
	24c	Describe and explain any amendments to information provided at registration or in the protocol.	No amendments to protocol/registration reported.
Support	25	Describe sources of financial or non-financial support for the review, and the role of the funders or sponsors in the review.	Funded by Asthma-Lung UK; grant AUK-PG-2019-419/ECSG24\45. Role of funder/sponsor not described. Funding (page 21).
Competing interests	26	Declare any competing interests of review authors.	Authors report a patent application for using miRNAs to diagnose asthma (named authors listed). Conflict of Interest (page 21).
Availability of data, code and other materials	27	Report which of the following are publicly available and where they can be found: template data collection forms; data extracted from included studies; data used for all analyses; analytic code; any other materials used in the review.	Manuscript analyses were based on previously published data and used public bioinformatic tools. Data Availability Statement (page 21).
